# Ancient Wheat Varieties and Sourdough Fermentation as a Tool to Increase Bioaccessibility of Phenolics and Antioxidant Capacity of Bread

**DOI:** 10.3390/foods11243985

**Published:** 2022-12-09

**Authors:** Tamara Dapčević-Hadnađev, Alena Stupar, Dušan Stevanović, Dubravka Škrobot, Nikola Maravić, Jelena Tomić, Miroslav Hadnađev

**Affiliations:** 1 Institute of Food Technology, University of Novi Sad, Bul. Cara Lazara 1, 21000 Novi Sad, Serbia; 2 Institute of Molecular Genetics and Genetic Engineering, University of Belgrade, Vojvode Stepe 9 444a, 11042 Belgrade, Serbia

**Keywords:** ancient wheat, antioxidant activity, bioaccessibility, breadmaking, sourdough

## Abstract

This study aimed to determine the impact of ancient wheat varieties (emmer, spelt and khorasan) and spontaneous sourdough fermentation on the bioaccessibility of total phenolic content (TPC) and the DPPH antioxidant capacity evolution during breadmaking and in vitro digestion. Sourdough and yeast-fermented modern wheat breads were used as controls. After 6 h of fermentation, the total titrable acidity of the sourdough increased from 139 to 167%. The wheat variety, type of fermentation and processing affected TPC, antioxidant activity and bioaccessibility. Antioxidant activity and TPC were reduced by dough mixing, increased after sourdough fermentation and slightly decreased or remained the same after baking. Although wheat flour had the highest TPC, the modeling of TPC kinetic revealed that emmer and spelt sourdough exhibited a higher bound phenolics release rate due to the higher acidity, which contributed to increased phenolics solubility. Although wheat bread, both before and after digestion, had the lowest TPC, especially the one prepared with yeast, high TPC bioaccessibilities and antioxidant activities after the digestion suggested that, except phenolics, digestion process improved the release of additional compounds with different bioaccessibility and biological activity. The results of this study proved that the application of sourdough fermentation can increase the potential of ancient wheats in the developing of functional bakery products.

## 1. Introduction

Common wheat (*Triticum aestivum*) represents one of the major cereal crops in the world with the annual global production in 2020 of approximately 760 million tons [1]. Wheat and wheat-based products are a good source of calories, essential amino acids, minerals and vitamins, beneficial phyto-chemicals and dietary fibers in human nutrition [2,3]. However, market demands in grain yield and desired wheat technological quality led to the creation of modern wheat varieties that are characterized by increased starch content which consequently results in a decrease of other components present in the grains, such as trace minerals [4]. Moreover, there is a rising health concern regarding the relationship between modern diet and lifestyle and increasing health issues such as obesity, diabetes type 2, various food allergies and intolerances, etc. Besides its role in satisfying certain quality attributes, consumers are also focused on the nutritional quality aspects of wheat-based products. In recent years ancient wheat varieties are generally recognized as healthier counterparts to modern wheat varieties. According to some studies, it was concluded that ancient wheat varieties had a higher content of protein, soluble dietary fiber, lipids, minerals and different bioactive compounds in comparison to modern wheat [5,6,7]. However, according to Shewry [8] there is not enough evidence to support the improved health benefits of ancient wheats which are influenced by genetically-determined differences in grain composition in comparison to modern wheat. Namely, these differences could be more presumable due to the effects of environmental conditions as well as interactions between environmental conditions and genotype. It is well known that wheat grains generally contain different phytochemicals with high biological activity. Polyphenols, plant secondary metabolites, are one of the important phytochemicals found in wheat. They include numerous compounds such as phenolic acids, coumarins, flavonoids, stilbenes and lignans [9]. According to Carter et al. [10] and Fardet [11], polyphenols exert more beneficial health effects such as antioxidants, anti-inflammatories, antimutagenics and anticarcinogenics. In addition, antioxidants have a large potential in the food production process, since they delay food deterioration, which occurs because of the oxidation, thus enhancing food preservation [12]. Dinelli et al. [9] investigated differences in polyphenol profile and content between 16 old and six modern wheat varieties. They concluded that the old wheat genotypes were characterized by the highest total polyphenol content and much higher number of total compounds and total isomers in comparison to modern wheat. Besides the total phenolic compound content, more attention should be addressed to its bioaccessibility and biological potential since a high proportion of phenolic acids is generally complexed with arabinoxylans and other indigestible polysaccharides, thus making them resistant to digestion in the upper gastrointestinal tract [13,14].

Several studies have indicated that applying processes such as sourdough fermentation can increase phenolic compound content and antioxidant activity of raw material [12,15,16]. During the sourdough fermentation process, the dough matrix is loosened and more extractable polyphenols are released. Moroni et al. [17] revealed that sourdough fermentation of buckwheat flour resulted in a significant decrease in phytate content and a twofold increase in polyphenols content compared to wheat-based bread. Moreover, according to Banu et al. [16] and Rizzello et al. [12] fermentation also results in the bioactive peptides release, thus increasing overall antioxidative activity. However, most of these studies are investigating the effect of inoculated lactic acid bacteria (LAB) fermentation on polyphenols content, while the impact of spontaneous sourdough fermentation induced by complex microflora from the air, water and flour is poorly exploited.

However, it is necessary to perform studies on the antioxidant activity and polyphenols bioaccessibility in sourdough after applying processes that are intended for human consumption such as breadmaking. The baking process influences polyphenols transformation and antioxidant activity in different ways. According to Abdel-Aal and Rabalski [18], the baking process may result in both a decrease or an increase in phenolic compound content as well as the antioxidant capacity of bread in comparison to dough which could be related to different processes occurring during the baking procedure. These processes include thermal degradation, polymerisation/depolymerisation, the release of bound phenolics and the development of numerous products of Maillard reaction. The decrease in total phenolic content as well as antioxidative activity of baked bread in comparison to sourdough could be related to loss/conversion processes that occur during the baking step and also due to the incorporation of additional ingredients during the dough breadmaking [16,19]. However, Abdel-Aal and Rabalski [18] and Gelinas and McKinnon [20] obtained higher content of phenolic acids and their derivatives and total phenolic content in wheat bread than in bread dough. They also reported that baking resulted in an increased amount of free phenolic acids but a decreased content of bound phenolic acids. According to Cheng et al. [21], heat stress in wheat could result in free phenolic acid increase due to the degradation of conjugated polyphenolic compounds. Opposite findings were presented by Holtekjølen et al. [22] and Menga et al. [23], who have demonstrated a decrease in free phenolic acids during the baking process. However, in their research, wheat bran or barley flour was also added in order to prepare pan and hearth bread.

In general, it remains unknown if ancient wheats are a better source of polyphenols and compounds with antioxidant capacity, if these compounds will be released with a different rate and in a different amount during spontaneous sourdough fermentation of both ancient and modern varieties, and what would be their fate after breadmaking with sourdough vs. yeast as a leavening agent and simulated in vitro gastrointestinal digestion. Therefore, the aim of the present study was to access polyphenols content and antioxidant activity transformation, as well as their bioaccessibility from the final product during the whole process of sourdough breadmaking; from wholegrain flour and the different phases of the sourdough fermentation process (0, 2, 4 and 6 h) to bread. The investigation was performed on both modern and ancient wheats (emmer, spelt and khorasan) in order to evaluate the impact of variety on the fate of polyphenols and antioxidant capacity during breadmaking. The role of sourdough on polyphenols and antioxidants distribution during processing was studied by comparing wheat sourdough bread with bakery yeast fermented bread.

## 2. Materials and Methods

### 2.1. Materials

Emmer, spelt and khorasan grains were donated from a local certified organic producer (Poljoprivredno gazdinstvo Spelta Jevtić, Bačko Gradište, Serbia), while Serbian winter wheat (*Triticum aestivum* L.) cultivar “Pobeda” served as a modern wheat variety. Interviews with a local organic producer revealed that emmer, spelt and khorasan seeds were non-certified, and were self-produced by farmers. All wheat samples were grown in the same location and weather conditions. Grains were stored for 6 months in the storage and then dehulled using a Heger’s large-scale friction de-huller (Herrenberg, Germany) and finally milled by a large-scale stone mill Osttiroler Getreidemühlen (Dölsach, Austria). Moisture, protein, total dietary fiber and ash content of the obtained flour samples were determined according to ISO 712:2012, ISO 20483:2013, AOAC 985.29 and ISO 2171:2012, respectively. The obtained results are presented in Table 1.

Compressed bakery yeast (Lesaffre, Belgrade, Serbia) and salt which were used in the breadmaking procedure were obtained from the local market.

### 2.2. Sourdough Preparation and Total Titrable Acidity (TTA) Measurement

Spontaneous fermentation of tested wheat flours was carried out using a backslopping procedure (every 24 h, 5 days, dough yield of 200) in a laboratory incubator (Friocell 111, MMM Medcenter Einrichtungen GmbH, München, Germany) at 25 °C. Mature sourdough was subsequently used as a “starter” instead of bakery yeast in the further breadmaking steps. 

In order to prepare the starter for breadmaking and further analysis, mature starter was mixed with flour and demineralized water in a 1:2:2 starter: flour: water ratio and incubated in a laboratory incubator at 25 °C for 6 h. This period of time was previously determined as a time needed for a preparation a mature sourdough starter. Dough samples were taken every 2 h during this 6 h of fermentation procedure. Samples at 0 h and 6 h were subjected to TTA measurements. Briefly, after the homogenization of dough samples (10 g) with distilled water (90 g), TTA was determined and expressed as the amount (mL) of 0.1N NaOH to achieve a pH of 8.5. Subsequently, dough samples (0 h, 2 h, 4 h and 6 h) for each flour variety (modern wheat, spelt, emmer and khorasan) were collected and frozen at −80 °C before the further freeze-drying procedure.

**Figure 1:**
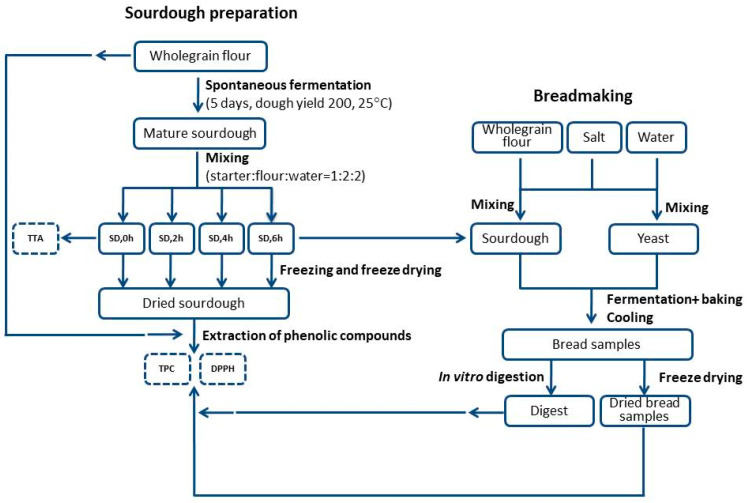
Scheme of experimental procedure used in this study. Abbreviations: SD—sourdough, TTA—total titrable acidity, TPC—total phenolic content, DPPH—antioxidant capacity according to DPPH method.

The scheme of the experimental procedure used in this study is illustrated in Figure 1.

### 2.3. Bread Making Procedure

Dough samples containing sourdough instead of bakery yeast as a leavening agent were prepared by replacing 25% of the flour with the appropriate type of sourdough in order to keep the total flour amount at 300 g required for the Farinograph mixing bowl (Brabender Technologie GmbH & Co., KG, Duisburg, Germany). An amount of 5.4 of salt and demineralized water up to 400 BU consistency were added and mixed in a Farinograph mixing bowl during the seven minutes. Control dough sample was prepared with 300 g of modern wheat flour, 7.5 g of bakery yeast, 5.4 g of salt and demineralized water in the amount required to achieve a consistency of 400 BU. 

Prepared dough samples were then left for fermentation in a cabinet at 30 °C during the 30 min, following by the punching down step. Dough samples were then rested and punched down an additional 3 times. Subsequently, 120 g of dough samples were hand-molded and transferred into teflon pans (L × W × H: 80 mm × 50 mm × 50 mm) for the final proofing step which was carried out at 30 °C and a relative humidity of 85% up to the optimum volume increase, which was 65 min for control dough and approximately 3 h for sourdough-containing samples.

Baking was performed in a modular deck oven (MD, Macpan SNS, Thiene, Italy) at 220 °C until a mass loss of 8% was reached. The obtained bread samples were then left to cool down to room temperature during the 2 h, cut to 10-mm thickness slices and placed in a laboratory freezer at –80 °C for further analysis. Two batches per each tested bread sample were prepared.

### 2.4. In Vitro Digestion of Bread Samples

To simulate the physiological process in the digestive tract (mouth, stomach, and small intestinal digestion sequentially), 5 g of ground fresh bread sample was subjected to GI digestion conditions in vivo (mouth, gastric and intestinal phases) according to assay described by Brodkorb et al. [24]. Briefly, bread samples were mixed with oral juice containing amylase (75 U/mL) at pH 7 and incubated in a shaking water bath (WNB 14, Memmert GmbH + Co.KG, Schwabach, Germany) for 2 min at 37 °C and 180 rpm. Further gastric juices containing pepsin solution (2000 U/mL) at a final pH 3 were added. Samples were incubated for 2 h at 37 °C and 180 rpm. Lastly, duodenal juices were incorporated containing bile salts (4.4 mg/mL) and pancreatin (100 U/mL), with pH 6.5–7 and incubated for 2 h at 37 °C and 180 rpm. At the end of the digestion, the enzyme reaction was stopped immediately by cooling the samples in ice, and the samples were kept at −20 °C until further analyses.

### 2.5. Freeze Drying

The lyophilization of previously freezed dough and bread samples (–80 °C) was carried out using an Alpha 1-2 LDplus freeze dryer (Martin Christ, Osterode am Harz, Germany) for 24 h for bread samples and 48 h for dough samples. Dried samples were homogenized using laboratory mortar, transferred in sealed polyethylene bags and stored at −18 °C prior further analyses.

### 2.6. Determination of Total Phenolic Content and Antioxidant Capacity

For the determination of total phenolic content and antioxidant activity gallic acid, Folin–Ciocalteu reagent, 2,2-diphenyl-1-picrylhydrazyl (DPPH) and 6-hydroxy-2,5,7,8-tetramethychroman-2-carboxylic acid (Trolox) were purchased from Sigma Aldrich, Germany. All other used chemicals were of analytical grade.

#### 2.6.1. Extraction of Free Phenolic Compounds

Prior to extraction, samples were milled in a kitchen blender in order to obtain homogenous sample. Ethanol was selected as a green solvent and as it was previously reported by several authors it shows the highest efficacy in the extraction of the total phenolic compounds from cereals [25,26,27].

Extraction from flour, dough and bread samples was performed using a two-step extraction assisted with the ultrasound. One gram of the sample was mixed with 20 mL of solvent (ethanol: water, 80:20, v:v) in extraction tubes using ultrasonic bath (Elma TI-H-15, Singen, Germany), operating at a frequency of 45 kHz for 10 min as was previously suggested in the research conducted by Milićević et al. [25]. The supernatants were separated, and the extraction was repeated with an additional 20 mL of fresh solvent. For all treatments, the temperature in the US bath was maintained at 25.0 ± 1.0 °C. After the extraction, supernatants were merged, centrifuged at 5000 rpm for 10 min (Eppendorf 5804 R, Hamburg, Germany) and filtered through Whatman paper. An aliquot of the supernatant (10 mL) was evaporated to dryness in a rotary evaporator at 35 °C. The dried extract was re-dissolved in ethanol to 1 mL volume. Extracts obtained in this way were further used for the determination of TPC and antioxidant activity. All experiments were performed in three replicates.

#### 2.6.2. Determination of Total Phenolic Content (TPC) in Flour, Dough, Bread and Bread after In Vitro Gastrointestinal Digestion

The total phenolic content in prepared extracts and digests was determined using colorimetric assay with Folin-Ciocalteu reagent [28]. The mixture was allowed to stand for 1 h for colour development and the absorbance at 765 nm was recorded using a UV 1800 spectrophotometer (Shimadzu, Japan). Gallic acid was used as a standard to establish the calibration curve for the quantification. The results were expressed as micrograms of gallic acid equivalents per gram of dry weight of sample (μg GAE/g dw). All experiments were performed in three replicates.

#### 2.6.3. DPPH Antioxidant Capacity of Flour, Dough, Bread and Bread after In Vitro Gastrointestinal Digestion

The antioxidant activity of samples was evaluated by DPPH radical scavenging activity assay. The absorbance of samples was measured at 515 nm after 60 min of standing in the dark. The antioxidant capacity was calculated from the Trolox calibration curve, and results were expressed as a Trolox equivalent (μmol TE/g dw). All experiments were performed in three replicates.

### 2.7. Kinetic Analysis

The kinetics of phenolic content and antioxidant capacity change during 6 h of sourdough fermentation were described by fitting a first-order kinetic model (Equation (1)) to the experimental data:

(1) $$\frac{A}{A_{0}}{=e}^{kt},$$

where *A* is total phenolic content or DPPH antioxidant capacity of sourdough, *A*_0_ indicates the initial value of the parameter *A*, *t* is the fermentation time and *k* is the rate constant for each wheat variety. The individual measurements were used instead of mean values of triplicate experiments and the results were expressed as mean values of *A*_0_ and *k*.

### 2.8. Bioaccessibility Determination

The bioaccessibility index of total phenols and antioxidant capacity was calculated according to Andrade et al. [29] using the Equation (2):

(2) $$\mathrm{BI}\left(\%\right)=\frac{D}{B}\times 100,$$

where *D* represents total phenolic content or antioxidant capacity after in vitro digestion, while *B* represents total phenolic content or antioxidant capacity before in vitro digestion. The bioaccessibility of total phenols and antioxidant capacity was evaluated in triplicate.

### 2.9. Statistical Analysis

Factorial ANOVA combined with post hoc Tukey HSD test (α = 0.05) were performed using Statistica 10 software (StatSoft Inc., Tulsa, OK, USA).

## 3. Results and Discussion

### 3.1. Changes in Total Titrable Acidity (TTA) during Sourdough Fermentation

In order to get insight into the growth and metabolic activity of the microbiota during spontaneous sourdough fermentation, progress of the TTA from initial (0 h) to final (6 h) fermentation point was monitored (Table 2). The most important compounds responsible for TTA increase during sourdough fermentation are lactic and acetic acids, with the prevalence of lactic acid content [16]. In this study, emmer and spelt sourdoughs were characterized by higher TTA values than khorasan and wheat, especially at the initial point (0 h), which was influenced by their flour composition rather than microbial metabolites formation. Katina et al. [30] have also showed that the final acidity of the sourdough is influenced by the characteristics of the rye flour, where rye flour with increase amylolytic activity is better fermented and allows getting more acidic sourdough. However, the TTA values rate increase during six hours of fermentation was higher for wheat (166.9%), khorasan (155.5%) and sourdough than spelt (143.3%) and emmer (139.2%), pointing to a more pronounced lactic acid production during the fermentation of the former. In general, the identification of lactic acid bacteria carried out by Coda et al. [31] has revealed the differences in biodiversity between different wheat varieties. While spelt flour harbored large microbial biodiversity, consisting of several species of lactobacilli and *W. confusa* and *Ped. pentosaceus/acidilactici*, emmer flour harbored a few species of lactic acid bacteria, with the predominance of *Lact. plantarum* isolates. According to Van der Meulen et al. [32], *L. fermentum* was found in stable wheat sourdough ecosystems, whereas it was not encountered in spelt sourdoughs.

### 3.2. Total Phenolic Content (TPC) Evolution during Baking

**Figure 2:**
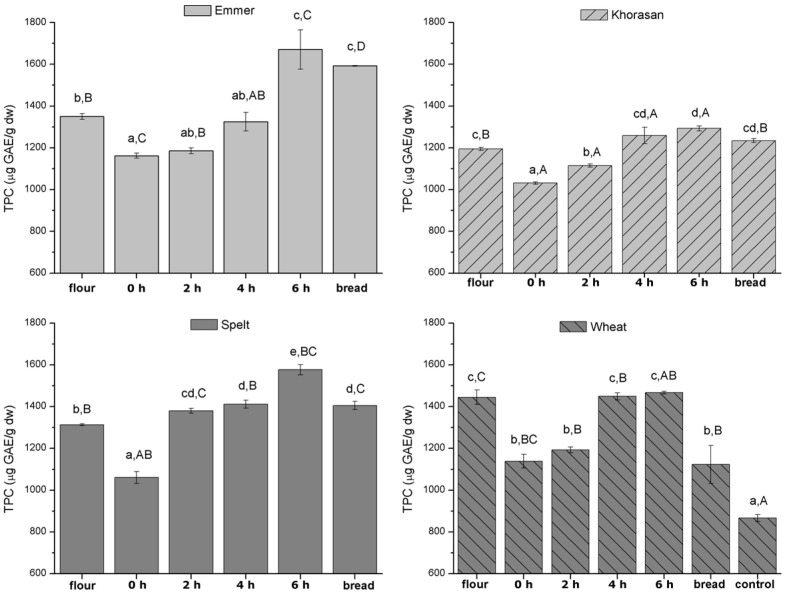
Evolution of total phenolic content during 6 h of sourdough preparation and breadmaking. Different lowercase letters indicate statistically significant differences within the group of the same wheat variety, while different uppercase letters indicate statistically significant differences between different wheat varieties in all stages individually (flour, different intervals of dough fermentation time and sourdough bread). Control refers to yeast fermented wheat flour bread, used as reference bread for comparison with the obtained sourdough breads.

The evolution of the phenolic content during the whole process of the sourdough breadmaking from different wheat varieties is presented in Figure 2. Total phenolic content of wholemeal (stone-ground) ancient wheat flours, which ranged from 1195 to 1350 μg gallic acid equivalents per gram of dry wholemeal sample, was slightly lower than that of wholewheat flour from the modern variety (1445 μg GAE/g dw). The TPC results obtained in this study are in agreement with the reported TPC of organic-solvent extractable phenolic compounds which are found to vary from 331 to 2620 μg GAE/g dry weight [3]. The opposite literature findings are available concerning polyphenols content in ancient and modern varieties. While some of the studies underline superior bioactivity of ancient wheat cultivars in terms of total phenolic content and antioxidant capacity [33,34], Shewry and Hey [3] have showed that ancient wheat species have similar contents of phenolic components to bread wheat, including total ferulic acid as the most abundant one.

In general, some of the authors underlined that variations in TPC are much more according to growth location, agronomic practices and environmental conditions than wheat variety [20], which explains the differences in studies comparing bioactive compounds in old and modern wheats. In addition, differences in the method employed for phenolics extraction (extraction solvent and extraction duration) could contribute to variations in the TPC content of wheat varieties.

Upon mixing the flour with water and part of the mature sourdough (Figure 2), a decrease in TPC occurred in all the wheat varieties. Although in the studies of Han and Koh [19] and Yu et al. [35], where the breadmaking procedure was performed with yeast as a leavening agent, our results were in agreement with the results of their investigations where antioxidant activities of phenolic acids slightly decreased during dough preparation, but increased during fermentation and baking process. The lower content of phenolics in dough compared to flour could be explained by the fact that dough mixing breaks disulfide bonds and creates thiol free radicals in gluten which reacts with reducing compounds in flour, such as phenolic acids, thus inhibiting disulfide crosslinking and free phenolic acids content [19]. 

During sourdough fermentation from 0 h to 6 h, a significant increase in TPC was recorded in all samples (Figure 2) which confirmed previous findings that sourdough biotechnology can represent a natural way to increase the bioactivity of dough [15,36]. The following mechanisms of lactic acid bacteria activity in increasing the phenolic compounds during sourdough fermentation were proposed: (i) the release of phenolic compounds due to hydrolysis of complexed and glycosylated forms as a consequence of the activity of LAB enzymes, (ii) hydrolysis and the release of bound phenolic compounds due to the activation of cereal enzymes (iii) the acidification of the system leading to improved solubilization of phenolic compounds and (iv) the conversion of phenolic acids to phenol and vinyl derivatives by LAB reductases and decarboxylases [36,37]. Although some studies have reported that type 2 sourdough fermentation (addition of starter cultures) is a more powerful tool to increase TPC content than type 1 (spontaneous) fermentation [12,16]. The results obtained in this study highlighted the importance of spontaneous fermentation in increasing polyphenols content.

Furthermore, the extent of the increase in TPC during 6 h of sourdough fermentation depended on the wheat variety, where emmer exhibited the highest rate constant (k) according to the results of the modelling of kinetics of phenolic content change during 6 h of sourdough fermentation (Table 3). In general, it could be noticed that although wheat flour was characterized with the highest initial TPC (*A*_0_ parameter in Table 3), the fastest released and/or conversion of phenolic acids contributed to higher content in TPC in emmer and spelt varieties. This could be related to the higher acidity of emmer and spelt sourdough than wheat which resulted in higher solubility of phenolic compounds. As already mentioned, in this study, the TTA of emmer and spelt sourdough was higher compared to the ones obtained in wheat (Table 2). In addition, the highest TPC increase rate observed during emmer sourdough fermentation could be explained by the fact that the content of total bound phenolic acids in emmer was found to be twice higher compared to that of spelt, soft wheat and durum wheat [38,39]. Benincasa et al. [38] have also revealed that p-coumaric acid accounted for about 50% of total bound phenolic acids. Therefore, emmer flour served as a pool of bound phenolic acids from which LAB and endogenous cereal enzymes hydrolyzed free forms that were detected after extraction with ethanol.

Upon baking, a decrease in TPC was observed compared to 6 h fermented sourdough in all the tested samples (Figure 2). However, in ancient wheat varieties, the content of TPC in sourdough bread was higher than in the flour, while the opposite effect was noticed in modern wheat bread and flour. Moreover, in yeast fermented bread, the content of TPC was the lowest, thus confirming the role of LAB metabolites in increasing phenolics content during breadmaking. The results obtained in this study were in accordance to the findings reported by Van Boxstael et al. [40] who observed a higher total phenol content (TPC) in breads prepared using ancient wheats than those from modern wheat. According to the literature findings, quite opposite effects concerning the impact of breadmaking on the fate of phenolic acids are observed. Banu et al. [16], Drakula et al. [36], Olojede et al. [41] and Yu et al. [35] reported lower phenolic acid content in bread compared to sourdough or yeast fermented dough, which they have attributed to the additional ingredients incorporated during bread dough preparation and loss/conversion of some phenolic acids, labile to baking. Li et al. [42] have revealed that other food processing techniques such as noodle and steamed bread production also influence TPC reduction. In contrast to this,, Gelinas and McKinnon [20] and Han and Koh [19] have observed higher antioxidant activity in bread than in fermented dough which was related to the release of the insoluble conjugated bound phenolic compounds due to increasing baking temperature accompanied by complex antioxidant melanoidin-products formation via the Maillard reaction during heating. In general, contrasting results could also be influenced with the differences in antioxidant compounds distribution between upper crust, bottom crust and bread crumb fractions [43].

### 3.3. DPPH Antioxidant Capacity Evolution during Breadmaking

**Figure 3:**
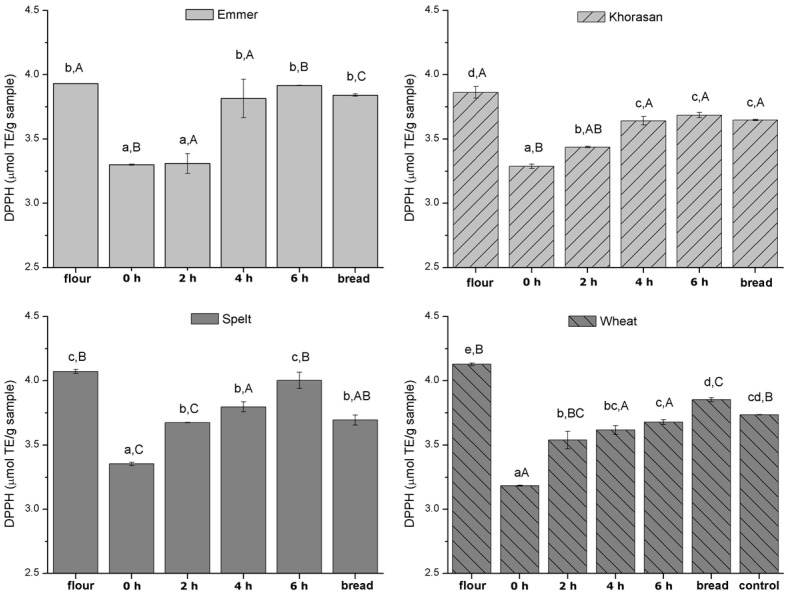
Evolution of DPPH antioxidant activity during 6 h of sourdough preparation and breadmaking. Different lowercase letters indicate statistically significant differences within the group of the same wheat variety, while different uppercase letters indicate statistically significant differences between different wheat varieties in all stages individually (flour, different intervals of dough fermentation time and sourdough bread). Control refers to yeast-fermented wheat flour bread, used as reference bread for comparison with the obtained sourdough breads.

The evolution of DPPH antioxidant capacity from flour to bread is presented in Figure 3, while the results of mathematical modelling (Equation (1)) of changes in antioxidant activity are summarized in Table 3. The DPPH radical scavenging activities of the samples were expressed as micromole Trolox equivalents per gram (µmol TE/g) of dried samples.

In general, the trend of DPPH antioxidant capacity change during breadmaking quite resembled the one observed for TPC content (Figure 2). However, the magnitude of the changes slightly differed. Moreover, in the case of the modern wheat variety, a significant increase in the antioxidant activity of both sourdough and yeast-fermented bread compared to sourdough was detected. This could be explained by the following: (i) the non-selectivity of Folin–Ciocalteu method in measuring the concentration of phenolic compounds, (ii) the nature of DPPH methods which besides antioxidant capacity of phenolic acids measures also the activity of other antioxidants present in the sample [36] or (iii) the formation of additional compounds with different bioaccessibility and biological activity in wheat bread during baking. In ancient wheat bread, a decrease in average scavenging ability during the bread-making process occurred, which could be attributed to the destruction of some phenolic compounds upon exposure to high temperatures used during baking. Due to the different polyphenol profiles in different wheat varieties [3], the extent of the decrease in DPPH scavenging abilities upon baking differed among different raw flour materials.

### 3.4. Bioaccessibility Assay of TPC and Antioxidant Capacity after Simulated In Vitro Digestibility

The bioaccessibility assay of TPC and antioxidant capacity, performed in this study, has been used to enhance the knowledge of the behaviour of these compounds. After the simulated in vitro digestion process, the TPC and antioxidant activity for the DPPH method ranged from 3788 to 5302 μg GAE/g dw and 14.26 to 15.17 μmol TE/g dw, respectively (Table 4), which were more than three-times higher values than the onces detected in bread samples before digestion. The digestion product of the wheat bread displayed the highest DPPH scavenging capacity, while emmer bread after digestion was characterized with the highest TPC, suggesting that the highest release of phenolic compounds did not contribute to the highest scavenger radical capacity. This revealed that sourdough fermentation and digestion process led to exposure or production of other new molecules with biological activity. The former statement could be supported with bioaccessibility values greater than 100% (Table 4), which suggested that additional compounds with different bioavailability and biological activity were released from the bread and/or metabolized/transformed from phenolic compounds in the intestinal environment [29]. Sánchez-Velázquez et al. [44] have also suggested that digestion increases the amount of phenolic metabolites (low molecular weight phenolics), enhancing their bioaccessibility, which could have a direct effect on their bioavailability and bioactivity. Moreover, unlike ethanol extract alkali hydrolysis during digestion results in additional release of bound phenolics from complexes with hydrolysable tannins, lignins, cellulose and proteins [35].

In addition, a study performed by Venturi et al. [33] revealed that protein digestibility was significantly affected by sourdough fermentation due to the proteolysis by LAB and the inactivation of some anti-nutritional factors such as trypsin inhibitor. Besides, acidification by lactic acid bacteria and the resulting reduction of disulfide bonds in gluten proteins increased the solubility of gluten proteins and activation of flour indigenous enzymes, hence making them more susceptible to proteolytic degradation [33] and the release of bioactive peptides which significantly contributed to the antioxidant capacity of wheat flour bread although it had a lower content of TPC (Figure 2). The study performed by Rizzello et al. [12] demonstrated the capacity of lactic acid bacteria to release peptides with antioxidant activity through the proteolysis of native cereal proteins.

The fact that the control bread, prepared in this study, exhibited the lowest TPC both before and after digestion suggested that sourdough fermentation represents a useful tool in increasing polyphenols release thus leading to creation of functional bakery products.

## 4. Conclusions

The results of this study revealed that sourdough fermentation, along with the incorporation of ancient wheat varieties, represents an important tool in the development of functional bakery products with increased antioxidant capacity and phenolics bioaccessibility. Sourdough fermentation improved the release of bound phenolics, even from the flours in which the initial free phenolics content was lower. This could potentially contribute to the avoiding of antioxidants addition in food formulation. In general, antioxidants are food additives which are negatively preserved by consumers. Therefore, their replacement would result in “clean label” product design without compromising its oxidative stability. Moreover, high phenolics bioaccessibilities and antioxidant activities after in vitro digestion suggested that, except phenolics, the digestion process improved the release of additional compounds with different bioaccessibility and biological activity, which is beneficial for health and well-being.

## Figures and Tables

**Table 1:** Characteristics of wheat flour varieties.

Sample	Wheat	Spelt	Emmer	Khorasan
Moisture	11.20 ± 0.09 ^b^	10.80 ± 0.07 ^a^	11.31 ± 0.11 ^b^	10.70 ± 0.06 ^a^
Protein (d.b.)	11.30 ± 0.32 ^a^	15.87 ± 0.37 ^b^	15.99 ± 0.30 ^b^	11.49 ± 0.43 ^a^
Total dietary fiber (d.b.)	10.45 ± 0.38 ^b^	10.10 ± 0.42 ^b^	9.58 ± 0.28 ^a^	9.85 ± 0.31 ^ab^
Ash (d.b.)	1.48 ± 0.08 ^a^	1.98 ± 0.06 ^b^	1.87 ± 0.04 ^b^	1.50 ± 0.07 ^a^

d.b.—on dry basis. The mean values ± standard deviation are not significantly different (*p* > 0.05) if they are followed by the same letters in the superscript.

**Table 2:** Total titrable acidity (TTA) of sourdough samples from different wheat varieties before (0 h) and after (6 h) fermentation.

TTA	0 h	6 h
Emmer	4.85 ± 0.085 ^d^	11.60 ± 0.042 ^h^
Khorasan	3.61 ± 0.007 ^a^	9.21 ± 0.041 ^e^
Spelt	4.46 ± 0.011 ^c^	10.61 ± 0.014 ^f^
Wheat	4.08 ± 0.042 ^b^	11.12 ± 0.056 ^g^

The mean values ± standard deviation are not significantly different (*p* > 0.05) if they are followed by the same letters in the superscript.

**Table 3:** Parameters of the first-order kinetic model of total phenolic content and antioxidant capacity change during 6 h of sourdough fermentation (*A_0_*—the initial value of phenolic content or antioxidant capacity, *k*—the rate constant).

Sample	TPC			DPPH		
	*A*_0_	*k*	r^2^	*A*_0_	*k*	r^2^
Emmer	980 ± 12.3 ^a^	0.127 ± 0.0055 ^c^	0.864 ± 0.0086	3.01 ± 0.028 ^a^	0.069 ± 0.0050 ^c^	0.907 ± 0.0356
Khorasan	958 ± 4.0 ^a^	0.080 ± 0.0036 ^a^	0.950 ± 0.0393	3.17 ± 0.010 ^bc^	0.040 ± 0.0008 ^a^	0.947 ± 0.0116
Spelt	993 ± 22.6 ^a^	0.114 ± 0.008b ^c^	0.868 ± 0.0193	3.21 ± 0.013 ^c^	0.056 ± 0.0047 ^bc^	0.957 ± 0.0236
Wheat	1026 ± 33.7 ^a^	0.096 ± 0.0074 ^ab^	0.887 ± 0.0268	3.12 ± 0.026 ^b^	0.045 ± 0.0001 ^ab^	0.868 ± 0.0080

The mean values ± standard deviation in the same column are not significantly different (*p* > 0.05) if they are followed by the same letters in the superscript.

**Table 4:** Total phenolic content (TPC) and DPPH antioxidant capacity after simulated bread in vitro digestion and bioaccessibility indexes (BI).

Sample	TPC		DPPH	
	Digestion Product (μg GAE/g dw)	BI (%)	Digestion Product (μmol TE/g dw)	BI (%)
Emmer	5302.1 ± 110.30 ^d^	333.0 ± 7.21 ^a^	14.26 ± 0.002 ^a^	371.2 ± 0.92 ^a^
Khorasan	4088.8 ± 35.80 ^b^	331.2 ± 0.42 ^a^	14.35 ± 0.032 ^a^	393.4 ± 0.35 ^c^
Spelt	4843.9 ± 15.59 ^c^	344.6 ± 3.60 ^a^	14.73 ± 0.046 ^b^	398.7 ± 5.36 ^c^
Wheat	4054.1 ± 8.66 ^b^	361.9 ± 28.49 ^a^	15.17 ± 0.009 ^c^	393.8 ± 1.39 ^c^

The mean values ± standard deviation in the same column are not significantly different (*p* > 0.05) if they are followed by the same letters in the superscript.

## Data Availability

Data is contained within the article.
